# Microalbuminúria e o Risco de Mortalidade em Pacientes com Insuficiência Cardíaca Aguda

**DOI:** 10.36660/abc.20220172

**Published:** 2022-04-07

**Authors:** Jerzy Beltowski

**Affiliations:** 1 Departamento de Fisiopatologia Universidade Médica de Lublin Lublin Polônia Departamento de Fisiopatologia - Universidade Médica de Lublin, Lublin – Polônia

**Keywords:** Insuficiência Cardíaca/fisiopatologia, Albuminuria/fisiopatologia, Diuréticos/uso terapêutico, Peptídeos Natriuréticos/uso terapêutico, Volume Sistólico

A insuficiência cardíaca (IC) é uma síndrome clínica crônica e progressiva resultante de anormalidades estruturais ou funcionais do coração. Os sintomas e sinais mais comuns da IC são dispneia, fadiga, congestão pulmonar e periférica ou edema e distensão da veia jugular. A prevalência de IC aumenta continuamente devido à melhoria do tratamento e à redução da mortalidade a curto prazo em pacientes com síndromes coronarianas agudas, doenças cardíacas congênitas, envelhecimento populacional e melhora da sobrevida de pacientes com insuficiência cardíaca já desenvolvida pela ampla aplicação de medicamentos modernos modificadores da doença e dispositivos.^1^

A insuficiência cardíaca aguda (ICA) é reconhecida quando os sintomas de IC aparecem no paciente sem história de IC prévia (IC *de novo*) ou quando os sintomas e sinais estão se exacerbando rapidamente em um paciente com IC previamente reconhecida (IC descompensada). A insuficiência cardíaca aguda é a causa mais comum de internações hospitalares não planejadas em pacientes idosos. A fisiopatologia de ambas as condições é semelhante, mas a IC *de novo* requer uma abordagem diagnóstica mais detalhada para encontrar a patologia subjacente. O tratamento inicial da ICA inclui diuréticos intravenosos e vasodilatadores de curta duração. A minoria dos pacientes com ICA apresenta choque cardiogênico associado a hipotensão e comprometimento grave da perfusão dos tecidos periféricos; o choque cardiogênico está associado a uma mortalidade muito maior do que a ICA sem choque. Enquanto o tratamento da IC crônica melhorou substancialmente as taxas de sobrevida, a evolução da ICA ainda é ruim, com altas taxas de mortalidade e reinternação hospitalar. A terapia atualmente usada é direcionada para reduzir a pré e pós-carga do coração e não visa a patologia subjacente específica em um determinado paciente, o que pode explicar a evolução insatisfatória dos resultados clínicos. Portanto, a terapia individualizada é muito apreciada, exigindo o estabelecimento de marcadores específicos.^2,3^

Neste número dos Arquivos Brasileiros de Cardiologia, Alataş et al. publicaram um interessante estudo sobre a microalbuminúria como marcador de mortalidade na ICA.^4^ Eles analisaram os dados de pacientes adultos admitidos no departamento de emergência com sinais e sintomas de ICA e aumento do peptídeo natriurético cerebral N-terminal (NT-proBNP). Os pacientes foram divididos em três grupos de acordo com a fração de ejeção do ventrículo esquerdo (FEVE): preservada (FEVE >50%, ICFEp), média (FEVE 40-49%, ICFEm) e reduzida (FEVE<40%, ICFEr) incluindo 213, 50 e 63 pacientes, respectivamente. As características demográficas e comorbidades foram coletadas no banco de dados do hospital. A albuminúria foi definida de acordo com a relação albumina/creatinina urinária (RAC): normoalbuminúria <30 mg/g, microalbuminúria 30-299 mg/ge macroalbuminúria >300 mg/g. A média de idade dos pacientes foi de 70,6 anos, sendo 53,3% do sexo feminino. Pacientes com ICFEr apresentaram valores mais elevados de NT-proBNP e RAC do que pacientes com ICFEm ou ICFEp. Não houve diferenças significativas na prevalência de normo, micro e macroalbuminúria entre os grupos com ICFEp e ICFEm; no entanto, tanto a micro como a macroalbuminúria foram mais frequentes na FHrEF do que nos dois grupos restantes. Não houve diferença no tempo de internação entre os grupos. A mortalidade intra-hospitalar foi maior na ICFEr (6,6%) do que na ICFEm (2,0%) ou na ICFEp (2,5%). De acordo com a análise multivariada, o NT-proBNP e a macroalbuminúria estiveram associados à mortalidade intra-hospitalar em todo o grupo. A microalbuminúria foi associada à mortalidade intra-hospitalar nos grupos ICFEr e ICFEm, mas não no grupo ICFEp. O risco de mortalidade hospitalar em pacientes com ICFEr foi 1,94 e 2,45 vezes maior naqueles com micro e macroalbuminúria, respectivamente, do que naqueles com normoalbuminúria. Micro e macroalbuminúria foram associadas a mortalidade 1,56 e 1,92 vezes maior em pacientes com ICFEm.

A albuminúria está associada à IC incidente na população geral e maior mortalidade entre os pacientes com IC estabelecida.^5^ No entanto, a relação entre microalbuminúria e subtipos de IC com FE preservada e reduzida é mais controversa. Ainda menos se sabe sobre a microalbuminúria como marcador na ICA. Em 2013 Koyama et al.,^6^ examinaram a evolução da RAC durante a internação em 115 pacientes com IC descompensada.^6^ Eles observaram uma diminuição na prevalência de microalbuminúria e da média da RAC entre os dias 1 e 7 de internação, e essa diminuição foi correlacionada com a diminuição do NT-proBNP e da bilirrubina sérica. Não houve diferença na FEVE entre os subgrupos com normo, micro e macroalbuminúria; no entanto, o NT-proBNP foi significativamente correlacionado com a RAC inicial. No entanto, a relação entre microalbuminúria e mortalidade não foi relatada. Recentemente, Wang et al.,^7^ examinaram a relação entre a concentração urinária de albumina e os resultados em 1.818 pacientes internados no hospital por ICAD. Os pacientes foram acompanhados por um período médio de 937,5 dias. A taxa composta de mortalidade, transplante cardíaco e implante de dispositivo de assistência ventricular esquerda foi 1,42 e 1,74 vezes maior em pacientes com micro e macroalbuminúria, respectivamente, do que naqueles sem albuminúria. Um modelo multivariado de regressão de Cox incluindo todas as variáveis significativamente associadas ao prognóstico demonstrou que micro e macroalbuminúria ainda eram os preditores significativos de mortalidade (taxa de risco 1,27 e 1,36, respectivamente). A análise de subgrupo demonstrou que a albuminúria predisse um risco maior de morte por todas as causas em pacientes com FEVE>40%, mas não naqueles com VE<40%. Assim, embora a microalbuminúria tenha sido associada a pior prognóstico em ambos os estudos,^6,7^ essa relação foi mais forte em pacientes com baixa FE no estudo de Alataş et al.,^4^ e naqueles com maior FE no estudo de Wang et al.,^7^ A razão para essa discrepância não é clara, entretanto, várias diferenças entre esses estudos devem ser destacadas. O estudo de Alataş et al.,^4^ incluiu pacientes com insuficiência cardíaca aguda (tanto de novo quanto descompensada), idade mais avançada (média de 70 anos) e TFGe mais baixa (média de cerca de 70 ml/min) e avaliaram a mortalidade hospitalar. Em contraste, Wang et al.,^7^ examinaram apenas pacientes com IC descompensada, idade mais jovem (mediana de 57 anos), TFGe mais elevada (média de cerca de 90 ml/min) e avaliaram o desfecho dentro do período mediano de quase 3 anos. Além disso, a RAC foi relatado no estudo de Alataş et al.,^4^ enquanto apenas a concentração absoluta de creatinina urinária foi medida por Wang et al.,^7^ Muito recentemente, Matsumoto et al.,^8^ demonstraram que o risco de reinternação precoce (dentro de 1 ano) era maior em pacientes com ICAD e micro ou macroalbuminúria do que naqueles com normoalbuminúria. Na análise multivariada, RAC e BNP foram os preditores independentes de reinternação. No entanto, o valor preditivo da RAC em subgrupos categorizados de acordo com FE não foi examinado.^8^

Em conclusão, a microalbuminúria surge como um novo marcador promissor em pacientes com insuficiência cardíaca aguda. O estudo de Alataş et al.,^4^ sugere que a microalbuminúria seja um preditor de mortalidade intra-hospitalar, principalmente naqueles com fração de ejeção reduzida. Embora a relação precise entre RAC, FE e outros marcadores, como peptídeos natriuréticos cardíacos, possa diferir dependendo das características dos pacientes e desfechos de interesse, este estudo^4^ e outros recentes^7,8^abrem uma nova área interessante de pesquisa e abordagem clínica individualizada.
