# Supplementary material for: Microalbuminúria e o Risco de Mortalidade em Pacientes com Insuficiência Cardíaca Aguda
Source: Arq Bras Cardiol. 2022 Apr 7;118(4):710–1. [Article in Portuguese] doi: 10.36660/abc.20220172 (PMC9007022; doi:10.36660/abc.20220172)
Supplement: Translation

# Translation: Microalbuminuria and the Risk of Mortality in Patients with Acute Heart Failure

**Authors:** Jerzy Beltowski

**Affiliations:** 1 Poland Departamento de Fisiopatologia - Universidade Médica de Lublin, Lublin – Poland

**Keywords:** Heart Failure/physiopathology, Albuminuria/physiopathology, Diuretics/therapeutic use, Natriuretic Peptides/therapeutic use, Stroke Volume

Heart failure (HF) is a chronic and progressive clinical syndrome resulting from structural or functional abnormalities of the heart. The most common symptoms and signs of HF are dyspnea, fatigue, pulmonary and peripheral congestion or edema and jugular vein distension. The prevalence of HF continuously increases due to improved treatment and reduced short-term mortality in patients with acute coronary syndromes, congenital heart disease, population aging and improved survival of patients with already developed heart failure by the widespread application of modern disease-modifying medications and devices.^1^

Acute heart failure (AHF) is recognized when symptoms of HF appear in the patient without the history of previous HF (de novo HF) or when symptoms and signs are rapidly exacerbating in a patient with previously recognized HF (decompensated HF). Acute heart failure is the most common cause of unplanned hospital admissions in older patients. The pathophysiology of both conditions is similar, but de novo HF requires a more detailed diagnostic approach to find the underlying pathology. The initial treatment of AHF includes intravenous diuretics and short-acting vasodilators. The minority of patients with AHF present with cardiogenic shock associated with low blood pressure and severely compromised perfusion of peripheral tissues; the cardiogenic shock is associated with much higher mortality than AHF without shock. Whereas the treatment of chronic HF has substantially improved survival rates, the outcome of AHF is still poor, with high mortality and hospital readmission rates. Currently used therapy is directed to reduce the pre and afterload of the heart and does not target the specific underlying pathology in a given patient, which may explain unsatisfactory progress of clinical outcomes. Therefore, individualized therapy is highly appreciated, requiring establishing specific markers.^2,3^

In this issue of the *Brazilian Archives of Cardiology*, Alataş et al. published an interesting study about microalbuminuria as a marker of mortality in AHF.^4^ They analyzed the data of adult patients admitted to the emergency department with signs and symptoms of AHF and increased N-terminal pro-brain natriuretic peptide (NT-proBNP). Patients were divided into three groups according to left ventricular ejection fraction (LVEF): preserved (LVEF >50%, HFpEF), mid-range (LVEF 40-49%, HFmrEF) and reduced (LVEF<40%, HFrEF) including 213, 50 and 63 patients, respectively. Demographic characteristics and comorbidities were collected from the hospital database. Albuminuria was defined according to urinary albumin-to-creatinine ratio (UACR): normoalbuminuria <30 mg/g, microalbuminuria 30-299 mg/g and macroalbuminuria >300 mg/g. The mean age of patients was 70.6 years, and 53.3% of them were females. Patients with HFrEF had higher NT-proBNP and UACR values than patients with HFmrEF or HFpEF. There were no significant differences in the prevalence of norm-, micro and macroalbuminuria between groups with HFpEF and HFmrEF; however, both micro and macroalbuminuria were more frequent in FHrEF than in the two remaining groups. There was no difference in length of hospital stay between groups. In-hospital mortality was higher in HFrEF (6.6%) than in either HFmrEF (2.0%) or HFpEF (2.5%). According to multivariate analysis, NT-proBNP and macroalbuminuria were associated with in-hospital mortality in the whole group. Microalbuminuria was associated with in-hospital mortality in HFrEF and HFmrEF groups but not in the HFpEF group. The risk of in-hospital mortality in patients with HFrEF was 1,94- and 2.45-fold higher in those with micro and macroalbuminuria, respectively, than in those with normoalbuminuria. Micro and macroalbuminuria were associated with 1.56- and 1.92-fold higher mortality in patients with HFmrEF.

Albuminuria is associated with incident HF in the general population and higher mortality among patients with established HF.^5^ However, the relationship between microalbuminuria and HF subtypes with preserved and reduced EF is more controversial. Even less is known about microalbuminuria as a marker in AHF. In 2013 Koyama et al.^6^ examined the evolution of UACR during hospitalization in 115 patients with decompensated HF.^6^ They observed a decrease in the prevalence of microalbuminuria and the mean UACR between days 1 and 7 of hospitalization, and this decrease was correlated with the decrease in NT-proBNP and serum bilirubin. There was no difference in LVEF between subgroups with normo, micro and macroalbuminuria; however, NT-proBNP was significantly correlated with baseline UACR. Nevertheless, the relationship between microalbuminuria and mortality was not reported. Recently, Wang et al.^7^ examined the relationship between urinary albumin concentration and outcomes in 1818 patients admitted to the hospital due to ADHF. The patients were followed for a median period of 937.5 days. The compound rate of mortality, heart transplantation, and left ventricular assist device implantation was 1.42- and 1.74-fold higher in patients with micro and macroalbuminuria, respectively, than in those without albuminuria. A multivariate Cox regression model including all variables significantly associated with prognosis demonstrated that micro and macroalbuminuria were still the significant predictors of mortality (hazard ratio 1.27 and 1.36, respectively). Subgroup analysis demonstrated that albuminuria predicted a higher risk of all-cause death in patients with LVEF>40% but not those with LV<40%. Thus, although microalbuminuria was associated with a worse prognosis in both studies.^6,7^ this relationship was stronger in patients with low EF in the study of Alataş et al.^4^ and in those with higher EF in the study of Wang et al.^7^ The reason for this discrepancy is unclear, however, several differences between these studies should be highlighted. The study of Alataş et al.^4^ included patients with acute heart failure (both de novo and decompensated), older age (mean 70 years) and lower eGFR )mean about 70 ml/min) and assessed in-hospital mortality. In contrast, Wang et al.^7^ examined only patients with decompensated HF, younger age (median 57 years), higher eGFR (mean about 90 ml/min) and assessed the outcome within the median period of almost 3 years. In addition, UACR was reported in the study of Alataş et al.^4^ whereas only absolute urinary creatinine concentration was measured by Wang et al.^7^ Very recently, Matsumoto et al.^8^ demonstrated that the risk of early (within 1 year) rehospitalization was higher in patients with ADHF and micro- or macroalbuminuria than in those with normoalbuminuria. In multivariate analysis, UACR and BNP were the independent predictors of rehospitalization. However, the predictive value of UACR in subgroups categorized according to EF was not examined.^8^

In conclusion, microalbuminuria emerges as a novel promising marker in patients with acute heart failure. The study of Alataş et al.^4^ suggests that microalbuminuria is a predictor of in-hospital mortality, especially in those with reduced ejection fraction. Although the precise relationship between UACR, EF and other markers such as cardiac natriuretic peptides may differ depending on the characteristics of patients and outcomes of interest, this study^4^ and other recent ones^7,8^ open a new interesting area of research and individualized clinical approach.
